# Supplementary material for: In transit: Cross-cultural differences in international students’ constructively aligned learning experience
Source: PLoS One. 2026 Apr 2;21(4):e0343971. doi: 10.1371/journal.pone.0343971 (PMC13046113; doi:10.1371/journal.pone.0343971)
Supplement: Attachment — Submitted filename: Response_to_Reviewers_auresp_2.docx [file pone.0343971.s003.docx]

**Response to Reviewers**

**PLOS ONE Submission: "In transit: cross-cultural differences in international students' constructively aligned learning experience"**

Dear Dr Wong and Distinguished Reviewers,

We are most grateful for your thoughtful and constructive feedback on this manuscript, and we deeply appreciate the time and expertise you invested in the review process.

**Response to Reviewer #1 (Dr Weijian Huang)**

Dear Dr Huang,

Thank you once again for your thoughtful and constructive feedback on our manuscript and for noting that the previous revisions had addressed your original concerns and improved the overall quality of the paper.

In your most recent comment, you kindly suggested reintroducing a clear conclusion signpost, for example by rewording the “Discussion” heading to “Discussion and Conclusion” and integrating the “Limitations and future directions” section into this closing part of the paper.

We fully agree with the importance of providing readers with an explicit structural cue for the conclusion.

In revising the manuscript, we chose to address this concern by structuring (i.e., re-arranging text without writing new text) the final part of the paper into a “Discussion” section (with the subheadings “Implications for theory” and “Implications for practice”) followed by a separate “Conclusion” section as a main heading. In the Discussion, we synthesise the key findings and elaborate the theoretical and practical implications of the study, whereas the standalone Conclusion section integrates the limitations and outlines directions for future research, rather than presenting these under an additional, separate heading. Our intention was to preserve a clear, conventional end point to the paper that functions as a strong signpost, while ensuring that both implications and future directions are tightly connected to the overall argument of the manuscript.

We hope that this structure meets the spirit of your suggestion, namely to avoid losing an explicit conclusion heading and to integrate limitations and future directions into the concluding part of the manuscript. We are very grateful for your detailed and insightful comments throughout the review process, which have significantly strengthened the clarity and contribution of the paper.

Yours sincerely,

Christian Stamov Roßnagel.

**Response to Reviewer #2**

Dear Reviewer 2,

Thank you very much for your thorough review and for your encouraging feedback on our manuscript. We are delighted that you consider the paper well-suited for publication at this stage and appreciate your recognition of the revisions we have undertaken.

We also thank you for your thoughtful remark regarding the sample size. As you note, the study is based on a modest sample, and we have therefore been careful to present the findings with appropriate caution, explicitly acknowledging the limitations for statistical power and generalisability, and outlining avenues for future research to address these constraints. We are grateful that you view this treatment of the limitation and future research directions as clear and satisfactory.

Finally, we are very pleased that you enjoyed the cross-cultural perspective offered by the paper. One of our central aims was to contribute to a more nuanced understanding of how constructive alignment operates in international and culturally diverse higher education contexts, and your positive evaluation is very encouraging in this regard.

Thank you again for your careful engagement with our work and for your supportive comments.

Yours sincerely,

Christian Stamov Roßnagel.
